# Commensal protist *Tritrichomonas musculus* exhibits a dynamic life cycle that induces extensive remodeling of the gut microbiota

**DOI:** 10.1093/ismejo/wrae023

**Published:** 2024-02-05

**Authors:** Ana Popovic, Eric Y Cao, Joanna Han, Nirvana Nursimulu, Eliza V C Alves-Ferreira, Kyle Burrows, Andrea Kennard, Noor Alsmadi, Michael E Grigg, Arthur Mortha, John Parkinson

**Affiliations:** Program in Molecular Medicine, The Hospital for Sick Children Research Institute, Toronto, ON, M5G 0A4, Canada; Department of Biochemistry, University of Toronto, Toronto, ON, M5S 1A8, Canada; Department of Immunology, University of Toronto, Toronto, ON, M5S 1A8, Canada; Department of Immunology, University of Toronto, Toronto, ON, M5S 1A8, Canada; Program in Molecular Medicine, The Hospital for Sick Children Research Institute, Toronto, ON, M5G 0A4, Canada; Department of Computer Science, University of Toronto, Toronto, ON, M5S 2E4, Canada; Molecular Parasitology Section, Laboratory of Parasitic Diseases, NIAID, National Institutes of Health, Bethesda, MD 20892, United States; Department of Immunology, University of Toronto, Toronto, ON, M5S 1A8, Canada; Molecular Parasitology Section, Laboratory of Parasitic Diseases, NIAID, National Institutes of Health, Bethesda, MD 20892, United States; Department of Immunology, University of Toronto, Toronto, ON, M5S 1A8, Canada; Molecular Parasitology Section, Laboratory of Parasitic Diseases, NIAID, National Institutes of Health, Bethesda, MD 20892, United States; Department of Immunology, University of Toronto, Toronto, ON, M5S 1A8, Canada; Program in Molecular Medicine, The Hospital for Sick Children Research Institute, Toronto, ON, M5G 0A4, Canada; Department of Biochemistry, University of Toronto, Toronto, ON, M5S 1A8, Canada; Department of Molecular Genetics, University of Toronto, Toronto, ON, M5S 1A8, Canada

**Keywords:** eukaryotic microbiota, protists, Tritrichomonas, microbiome, pathogen

## Abstract

Commensal protists and gut bacterial communities exhibit complex relationships, mediated at least in part through host immunity. To improve our understanding of this tripartite interplay, we investigated community and functional dynamics between the murine protist *Tritrichomonas musculus* and intestinal bacteria in healthy and B-cell-deficient mice. We identified dramatic, protist-driven remodeling of resident microbiome growth and activities, in parallel with *Tritrichomonas musculus* functional changes, which were accelerated in the absence of B cells. Metatranscriptomic data revealed nutrient-based competition between bacteria and the protist. Single-cell transcriptomics identified distinct *Tritrichomonas musculus* life stages, providing new evidence for trichomonad sexual replication and the formation of pseudocysts. Unique cell states were validated *in situ* through microscopy and flow cytometry. Our results reveal complex microbial dynamics during the establishment of a commensal protist in the gut, and provide valuable data sets to drive future mechanistic studies.

## Introduction

Research into host-associated microbiomes has established the critical role of gut bacteria in health and disease [1, 2]. Emerging data supports an appreciable presence of gut-dwelling protists with host immunomodulatory capabilities and influence over resident bacterial communities, suggesting these microbes have the capacity to shape the gut environment [3–7]. Studies in this discipline are in their infancies, however, and lack understanding of both the ability of protists to colonize and persist and protist:bacterial cross-talk in the context of host immunity.

The protist *Tritrichomonas musculus* (*Tmu*) is a common resident of the murine gut, and a relative of prevalent human trichomonads whose role in gut health is unclear [6, 8–10]. *Tmu* establishes chronic asymptomatic infections, resulting in elevated baseline intestinal immune activation and structural remodeling of the intestinal epithelium [6, 11–13]. Protist-produced succinate furthermore stimulates a Th2-based immune response that restricts infection by enteric parasites [12–14]. Although the sustained inflammation associated with *Tmu* confers protection to mice against pathogenic bacteria, it can increase susceptibility to T-cell-driven colitis and tumorigenesis, suggesting *Tmu* behaves as a pathobiont. Evidence shows its presence influences gut bacterial composition, and that conversely *Tmu* engraftment may be modulated by bacterial taxa such as *Bifidobacterium* spp. through unknown mechanisms [15]. These microbial interactions may in part be driven through host immunoglobulins (Ig) A and M, antibodies secreted by B cells and elevated in response to *Tmu* [6, 13]. Both bacteria and protists have known effects on B-cell antibody responses, but it is not well understood how host B cells modulate cross-kingdom microbial interactions [6, 16, 17].

Here we track *Tmu* activities during the first 28 days of gut colonization, together with its interactions with resident bacteria in the context of healthy WT and B-cell-deficient (*muMt^−/−^*) mice through 16S rRNA sequence surveys and microbial transcriptomics. We show that *Tmu* colonization dramatically remodels bacterial composition and function, concurrent with shifts in protist metabolism and virulence factor expression, and that these changes are accelerated in the absence of host B cells. We provide detailed transcriptomic analyses suggesting cross-kingdom competition for key dietary nutrients. Finally, we conduct single-cell level characterization of the *Tmu* life cycle and validate cell stages *in situ* through fluorescence-based labeling of stage-specific transcripts. Our data reveal B-cell-modulated co-adaptation between resident bacteria and a newly colonizing protist, and provide valuable data sets to drive future mechanistic studies.

## Materials and methods

### Mice

C57Bl/6J and littermate B6.129S2-*Ighm^tm1Cgn^*/J (*muMt*^−/−^) female mice (Jackson Laboratory, Bar Harbor, ME, USA) were maintained in specific pathogen free (SPF) conditions, and mice of the same genotype were cohoused prior to the experiment. Experiments were conducted at 6–8 W of age. For single-cell protist sequencing, 12 W germ-free (GF) female C57Bl/6 mice (Taconic Biosciences, Germantown, NY, USA) were housed in the GF facility until the day of analysis, when they were transported to SPF conditions. Mice conventionalized with a microbiome received a bacterial suspension through oral gavage derived from two C56Bl/6J fecal pellets in PBS, and were maintained under SPF conditions for 4 weeks prior to protist colonization. Animals were housed in a closed caging system and provided with irradiated chow (Envigo Teklad 2918), non-acidified water (reverse-osmosis and UV-sterilized) with a 12-h light/dark cycle. Following protist colonization, animals were housed in separate cages. Animal experiments were approved by the Local Animal Care Committee at the Faculty of Medicine, University of Toronto (animal use protocol 20012400).

### Protist colonization

Purification of *Tmu* was performed as previously described [6]. Briefly, caecal contents of a colonized C57Bl/6J mouse were filtered through a 70-μm cell strainer, washed with PBS, and protists were collected from the interphase after density centrifugation through 40% Percoll overlaid on 80% Percoll. Cells were sorted into PBS on a BD Influx Cell Sorter (BD Biosciences, Franklin Lakes, NJ, USA) using the 100-μm nozzle at 27 psi at 4°C, with >99% purity. Mice were orally gavaged with 2 million *Tmu* cells. Protists were quantified using a hemocytometer.

### Genome assembly

Genomic DNA was extracted from sorted protists using the MagAttract HMW DNA Kit (QIAGEN, Hilden, Germany), and sequenced on two SMRT cells using a Sequel System (PacBio, Menlo Park, CA, USA). One million reads were assembled into contigs and polished with 8.6 million 300-bp paired-end reads generated on a MiSeq system (Illumina, San Diego, CA, USA) as detailed in Supplementary Methods. Gene models were predicted with the Maker v2.31 pipeline and annotated using InterProScan v.5.30-69.0, HmmerWeb v.2.41.2 and Architect [18–21]. Genes encoding adhesins, meiosis, and cell cycle-related proteins were identified based on sequence homology with *Trichomonas vaginalis* proteins retrieved from TrichDB [22–24]. The genome assembly is accessible at: https://github.com/ParkinsonLab/Tritrichomonas-murine-microbiome-interactions.

### Phylogenetic analysis

Parabasalid ribosomal internal transcribed spacer (ITS) sequences were downloaded from GenBank (see Supplementary Methods) and compared with *Tmu* ITS [25]. Multiple sequence alignments were generated using MUSCLE, and an unrooted phylogeny was constructed using the Maximum-likelihood method with a Tamura–Nei model in the MEGA-X software [26–28]. Bootstrap values were generated using 1000 replicates.

### Single-cell transcriptomics

Single-cell RNA sequencing (scRNA-Seq) was carried out for protists isolated from a GF and conventionalized mouse four weeks post colonization. Protists were purified from caecal contents and immediately transferred on ice for library preparation using Drop-seq technology and sequenced on a NextSeq 500 system (Illumina, San Diego, CA, USA) [29]. Reads were processed using Drop-seq Tools v.1.13 and aligned to the genome assembly using STAR v.2.5.3a [30, 31]. Cells were grouped using graph-based clustering and visualized via UMAP in Seurat v4 [32–34]. Differentially expressed (DE) genes were identified using the FindAllMarkers function, and functional enrichments were determined based on overrepresentation of pathway enzymes (based on Enzyme Classification (EC) annotations) as defined by KEGG using the hypergeometric test, or GO terms using the topGO package and the Fisher’s Exact test [35, 36]. Enrichments of meiosis, G1/S, and G2 phase genes were scored with the AddModuleScore function and evaluated using two-sided Wilcoxon rank-sum tests. Benjamini–Hochberg correction was applied for multiple testing [37]. Heatmaps were generated using pheatmap 1.0.12 and Ward.D2 clustering [38].

### 16S rRNA sequencing and metatranscriptomics

**Figure 1:**
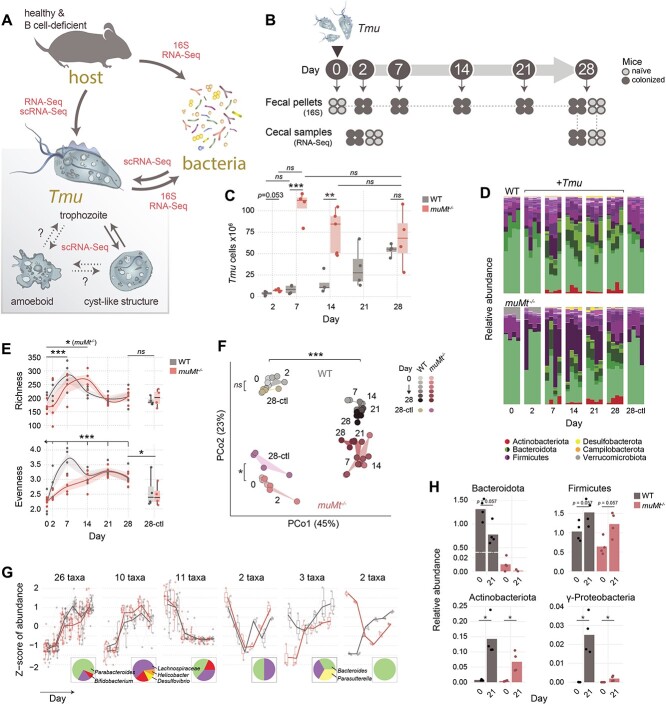
*Tmu* colonization drives bacterial diversification. (A) Schematic of microbial interactions explored in the present study using 16S rRNA sequencing, metatranscriptomics (RNA-Seq), and/or single-cell transcriptomics (scRNA-Seq). (B) Experimental design. Groups of four WT and *muMt^−/−^* mice were colonized with *Tmu*. Fecal pellets and caecal samples were collected at indicated timepoints for 16S rRNA and metatranscriptomic profiling. (C) Expansion of *Tmu* cells in WT and *muMt*^−/−^ mouse caeca over 28 days of colonization (*n* = 4 or 5 per group). Differences were tested using a two-sided *t*-test. (D) Relative abundance of bacterial taxa determined through 16S rRNA sequencing of mouse fecal pellets from groups of WT and *muMt*^−/−^ mice sampled over 28 days of colonization, and groups of uninfected controls sampled on day 28 (*n* = 4 per group). Colors represent genus-level abundances within indicated phylum color groups. (E) Bacterial richness (observed OTUs) and evenness (Shannon Index) over the course of *Tmu* colonization. Differences between timepoints were evaluated using LME, and between colonized and naïve mice at day 28 using two-way ANOVA. (F) Principal coordinate analysis of Bray–Curtis dissimilarities between samples. Significance was tested using permutational analysis of variance with adonis. (G) Patterns of bacterial abundance changes during *Tmu* colonization in WT (gray) and *muMt*^−/−^ (red) mice. Pie charts show the taxonomic make up of each cluster. (H) Phylum-level bacterial abundance before (at day 0) or 21 days after protist colonization, determined through qPCR using clade-specific primers. Abundances were normalized to Ct values obtained with universal 16S rRNA probes. Bars show means. Significance was evaluated using the Wilcoxon rank-sum test: ^*^P < .05, ^*^^*^P < .01, ^***^P < .001, ns not significant.

Groups of four WT or *muMt*^−/−^ mice were infected with protists for 16S rRNA and gene expression profiling. A schematic of the experiment is presented in Fig. 1B.

For 16S rRNA profiling, mouse fecal pellets were collected from protist-colonized WT or *muMt^−/−^* mice at days 0, 2, 7, 14, 21, and 28, and uninfected control mice at day 28, and stored at −80°C. DNA was extracted using the DNeasy PowerSoil Kit (QIAGEN, Hilden, Germany) according to manufacturer instructions, with 5-min bead beating using the FastPrep-24 homogenizer (MP Biomedicals, Santa Ana, CA, USA) at 5 M/s. Amplicons were generated using B969F and BA1406R primers and paired-end sequenced (250 bp) on a MiSeq system (Illumina, San Diego, CA, USA) [39]. Reads were preprocessed using Dada2 within QIIME2, subsequently *de novo* clustered to 97% operational taxonomic units (OTUs), and classified with a classifier trained on V6–V8 sequences from the SILVA v138 database [40–42]. Diversity and multivariate analyses were performed with Phyloseq 1.26.1 [43] and vegan v.2.5 [44]. Average alpha diversities were evaluated from 100 independently rarefied data sets to the minimum read depth, using linear mixed effects models with lme4 or a two-way ANOVA [45]. Beta diversities were determined for rarefied OTU data and tested using the adonis function in vegan. Differentially abundant taxa were identified with DESeq2 v.1.22.2 and grouped by abundance pattern with DEGreport v.1.26.0 [46, 47]. Networks were constructed in Cytoscape v3 based on Kendall rank correlations (minimum 0.7) of interbacterial and *Tmu*-bacterial abundances over time, calculated in DEGReport; clusters were defined using the Markov Cluster Algorithm [47–49].

For metatranscriptomics, caecal contents were collected from both colonized and naïve mice at days 2 and 28 of the experiment. RNA was extracted using TRIzol (Invitrogen, Carlsbad, CA, USA) and homogenized with 0.1-mm Zirconia beads (BioSpec Products, Bartlesville, OK, USA) using a TissueLyser (QIAGEN, Hilden, Germany), followed by the PureLink RNA Mini Kit (Invitrogen, Carlsbad, CA, USA) according to the manufacturer TRIzol Plus Total Transcriptome Isolation protocol. rRNA was depleted using the QIAseq FastSelect rRNA HMR and 5S/16S/23S kits (QIAGEN, Hilden, Germany), and libraries were paired-end sequenced (150 bp) on NovaSeq 6000 (Illumina, San Diego, CA, USA). Reads were processed and annotated using MetaPro, and protist sequences were mapped to the *Tmu* genome assembly using STAR v. 2.5.3a [31, 50]. Proteins with iron-related functions were predicted using FeGenie [51]. Bacterial cell division and cell wall biogenesis machinery were predicted through sequence similarity with previously described *Escherichia coli* MG1655 proteins using DIAMOND v.0.9.22 [52, 53]. DE was evaluated with DESeq2 v.1.22.2 [46]. Because of differences in bacterial read depths in protist-colonized and uninfected mice, bacterial gene counts were rarefied to 600 000 reads per sample, and DE was averaged from 15 independent rarefactions. Functional enrichment was evaluated using KEGG pathway or GO term overrepresentation as above.

### Quantitative polymerase chain reaction

Quantitative polymerase chain reaction (qPCR) was performed on 10-ng fecal DNA using the PowerTrack SYBR Green Master Mix (Applied Biosystems, Waltham, MA, USA) with 400-nM primers targeting 16S rRNA of Actinobacteriota (920F, 1200R), Bacteroidota (798F, 967R), Firmicutes (934F, 1060R), or Gammaproteobacteria (1080F, 1202R) [54] (see Supplementary Methods) and the following cycling conditions: 95°C for 2 min, 40 cycles of 95°C for 15 s and 60°C for 60 s. Relative abundances were normalized to the universal 16S rRNA gene, and calculated using the delta–delta Ct method.

### RNA FISH-flow cytometry

Four million protists were stained with Zombie Aqua viability dye (BioLegend, San Diego, CA, USA) in PBS in the dark (1:1000, 15 min, 4°C), washed twice with PBS, and the remaining steps were performed as described in the Stellaris RNA-FISH protocol for cells in suspension. After hybridization, cells were sorted on a BD LSR Fortessa X-20 cell analyzer (BD Biosciences, Franklin Lakes, NJ, USA). To test for pseudocyst formation, protists were isolated from the mouse caecum or colon and stained with wheat germ agglutinin (WGA)-FITC (Sigma Aldrich, St. Louis, MO, USA) in FACS buffer (PBS, 2% FBS, 5-mM EDTA) in the dark, either immediately or after 1–3 days *in vitro* anaerobic culturing. Cytospins were visualized using a Zeiss AXIO Observer microscope (Carl Zeiss AG, Jena, Germany).

### RNAscope

RNAscope was performed as per the RNAscope Multiplex Fluorescent Reagent Kit v2 (Advanced Cell Diagnostics, Newark, CA, USA) protocol. Approximately 0.5-cm caecum sections were excised from *Tmu*-colonized mice, fixed in 10% formalin, and prepared as 7-μm paraffin sections, as detailed in Supplementary Methods. Antigen target retrieval was conducted at 99°C under the 15-min standard procedure. A barrier was created around sections using an ImmEdge pen (Vector Laboratories, Burlingame, CA, USA), samples were treated with protease, and labeled with TSA Plus Fluorophores Fluorescein and Cyanine 3 hybridized against TMU_00005724 and TMU_00016742 probes, respectively. Samples were visualized using a Zeiss AXIO Observer microscope (Carl Zeiss AG, Jena, Germany).

### Transmission electron microscopy

Protist pellets were prepared using the Embed 812 resin kit (Electron Microscopy Sciences (EMS), Hatfield, PA, USA) [55]. Briefly, samples were fixed with 4% paraformaldehyde, 1% glutaraldehyde in phosphate buffer (PB, 0.1 M, pH 7.2), followed by 1% OsO4 in PB in the dark. After washing with PB, samples were dehydrated in a 30%–100% gradient ethanol series, infiltrated with increasing amounts of Embed 812 resin in propylene oxide, and cured in molds at 65°C. 80-nm sections were prepared with a Reichert Ultracut E microtome (Leica, Wetzlar, Germany) on 300 mesh copper grids (EMS), and counter stained with saturated 5% uranyl acetate, followed by Reynold’s lead citrate (EMS). Sections were imaged using a Talos L120C transmission electron microscope (Thermo Fisher Scientific, Waltham, MA, USA). See Supplementary Methods for details.

## Results

### *Tmu* colonization drives diversification of the intestinal microbiome

Colonization with *Tmu* induces profound changes in the mouse gastrointestinal tract, including the immune landscape known to maintain gut microbial homeostasis [6, 17]. In light of this, we characterized the impact of *Tmu* engraftment on the local bacterial community in a healthy and immune-impaired host (Fig. 1A). We tracked bacterial composition and activities in WT and B-cell-deficient (*muMt*^−/−^) C57Bl/6 mice through 16S rRNA surveys of mouse fecal pellets and metatranscriptomics of caecal microbiota (Fig. 1B, Supplementary Data 1–3) during the first 28 days of colonization. Protist expansion was accelerated in *muMt*^−/−^ mice, reaching its maximum level within the first 7 days, whereas in WT mice, expansion steadily increased until day 28 (Fig. 1C).

*Tmu* dramatically altered gut bacterial composition in both WT and *muMt*^−/−^ mice relative to days 0 and 28 naive mice (Fig. 1D). Bacterial richness increased during the first week of colonization, with a delay in *muMt*^−/−^ mice, before receding to near day 0 levels (Fig. 1E). Evenness increases persisted to day 28, reflecting expansions in multiple taxa. Although WT and *muMt*^−/−^ mouse communities differed initially, they converged toward similar profiles after protist infection (Fig. 1F). Bacteria in day 28 control mice were most similar to day 0 naïve bacterial compositions (prior to *Tmu* exposure) rather than day 28 colonized mice, underlining a protist-driven shift. We observed six distinct bacterial abundance patterns in *Tmu*-colonized mice (Fig. 1G). The majority changed congruently in colonized WT and *muMt*^−/−^ mice, and included taxa that increased throughout (e.g. *Bifidobacterium* and *Parabacteroides*), peaked at day 21 (e.g. *Helicobacter*, *Desulfovibrio*, and *Lachnospiraceae*), or decreased after *Tmu* colonization; five taxa exhibited discordant patterns (e.g. *Parasutterella* and *Bacteroides*). Details from the differential abundance analysis may be found in Supplementary Data 4. Changes in bacterial abundances were validated via qPCR (Fig. 1H). To investigate the patterns further, we constructed microbial interaction networks from correlations of changes in *Tmu* and bacterial abundances over time (Supplementary Fig. S1). These networks recapitulate our findings above (Fig. 1) with the generation of two dominant clusters. Cluster 1 comprises *Tmu* and positively correlated taxa, consistent with the first two patterns of bacterial abundance (Fig. 1G). Taxa associated with cluster 2 are negatively correlated with *Tmu* and other members of cluster 1, and are dominated by bacteria with abundance patterns 3 and 4 (Fig. 1G, Supplementary Fig. S1).

### *Tmu* drives changes in bacterial metabolism

**Figure 2:**
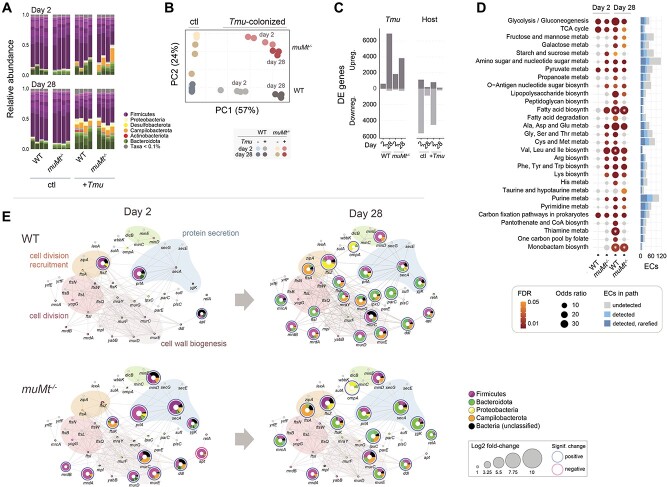
Bacterial gene expression is altered in the presence of the protist. (A) Taxonomic profiles of putative bacterial mRNA reads. (B) Principal component analysis of bacterial gene expression. Circles denote samples isolated from individual mice, colored by host B-cell status, presence of *Tmu* and timepoint, as shown. Expression values are based on DESeq2-variance stabilized counts. (C) Numbers of significantly upregulated or downregulated bacterial genes between protist-colonized and naïve mice, or due to host B-cell status, grouped as indicated (*n* = 3 or 4). (D) Bacterial metabolic pathway enrichment associated with *Tmu* colonization. Enrichment was determined by the overrepresentation of EC terms in KEGG-defined pathways within DE genes. Total ECs detected in the experiment and in the rarefied gene expression matrix used in the analysis are indicated in the bar chart to the right. Significantly enriched pathways are represented as colored dots: gray indicates ns, and the gradient from yellow to red represents decreasing *P* values, beginning from *P* < .05. Sizes of dots represent odds ratios. Significance was calculated using Fisher’s exact tests. Asterisks indicate infinite odds ratios. (E) Network of cell division, cell wall biogenesis, and secretory genes, based on a previously determined *E. coli* protein network [52]. Colored nodes represent protein homologs identified in the metatranscriptomic data and edges are previously described protein–protein or functional interactions. Nodes in gray represent undetected genes. Genes significantly up- and downregulated are denoted with blue and red borders, respectively. Node sizes correlate with log2 fold-changes between *Tmu*-colonized and naïve mice. Colors within node pie charts indicate proportions of gene expression assigned to various taxa, as shown. Colors encircling groups of proteins define functional modules.

To investigate the impact of *Tmu* on community functions, we performed bulk RNA sequencing of naïve and colonized mouse caecal contents at days 2 and 28, early and chronic stages of infection (Supplementary Fig. S2, Supplementary Data 3). We identified 835 million bacterial reads, 741 million in naïve, and 94 million in colonized mice. *Tmu* colonization was associated with blooms in Campilobacterota, Proteobacteria, and Bacteroidota activities (Fig. 2A). The increase in gene expression of the former two was accelerated in *muMt^−/−^* mice, accounting for 14% of reads by day 2 compared with 4% in WT mice and 1%–3% in naïve mice, suggesting B cells mediate the shift in bacterial gene expression consequent to protist colonization. The majority of these reads mapped to *Helicobacteraceae* (especially *Helicobacter ganmani* and *Helicobacter rodentium*) previously associated with enteric inflammation and exacerbated colitis [6, 13, 56].

Principal component analysis of bacterial gene expression recapitulated our 16S rRNA findings (Fig. 2B). *Tmu* colonization resulted in overall upregulation of bacterial genes, and although already apparent in *muMt*^−/−^ mice at day 2, B-cell-associated differences converged over time (Fig. 2C). Pathway analysis revealed upregulation of multiple metabolic pathways including glycolysis, metabolism of amino acids, and biosynthesis of peptidoglycans, polysaccharides, O-Antigen sugars, and fatty acids (Fig. 2D). Metabolic activity shifted from Firmicutes at day 2 to Bacteroidota, Campilobacterota, and Proteobacteria by day 28 (Supplementary Figs S3 and S4). The expansion of Proteobacteria, particularly in the expression of succinyl-CoA synthetase that is typically suppressed under anaerobic conditions, may indicate increased exposure to oxygen characteristic of gut inflammation and dysbiosis (Supplementary Fig. S3) [57].

Given the central role of these pathways in bacterial growth, we examined the expression of homologs to 44 genes associated with bacterial cell division and cell wall biosynthetic machinery in *E. coli* (Fig. 2E) [52]. Patterns were consistent, with earlier upregulation of genes in *muMt*^−/−^ colonized mice and a shift from Firmicutes to Bacteroidota, Campilobacterota, and Proteobacteria in response to *Tmu* by day 28. Day 2 bacteria from *muMt*^−/−^ mice exhibited increased chromosome segregation (e.g. *ftsK* and *ftsZ*), cell elongation (e.g. *mrdA* and *mrdB*), and peptidoglycan biogenesis (e.g. *murCDE*). By day 28, these genes were upregulated in bacteria across both hosts, together with genes involved in protein secretion and porin *ompA*, which modulates infection and host immunity in Gram-negative bacteria. Collectively, these data demonstrate substantial protist-mediated changes in bacterial activities, and an ecosystem shift to promote growth of Gram-negative taxa.

### *Tmu* activity changes in a B-cell-dependent manner

**Figure 3:**
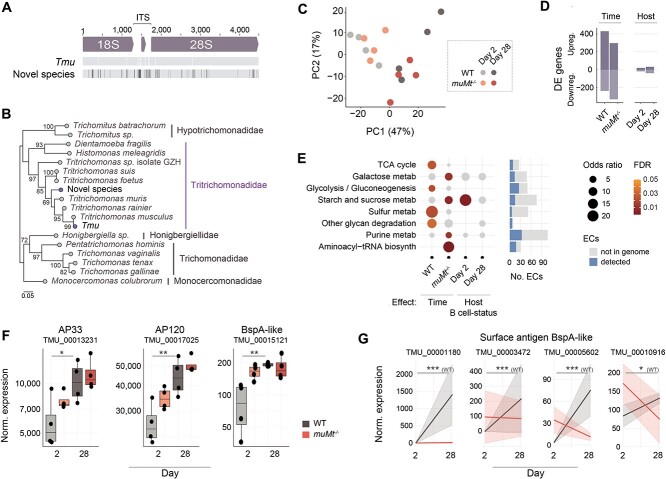
Protist gene expression changes during colonization of WT and *muMt*^−/−^ mice. (A) Graphical depiction of aligned rRNA sequences identified in the protist metagenome assembly. The dominant sequence was synonymous with *Tmu* and mapped to 81% of reads; the second distinct sequence mapped to 19% of reads. Sequences were aligned using MUSCLE. Mismatches in the sequence of the novel organism to *Tmu* are indicated in black. (B) Phylogeny of the two ribosomal ITS sequences identified in the metagenome. An unrooted tree was constructed from aligned sequences using the Maximum-likelihood method. Scale = number of SNPs per site. Bootstrap values > 65% are shown. (C) Principal component analysis of protist gene expression patterns at days 2 and 28, in WT or *muMt*^−/−^ mouse caeca. (D) Numbers of DE genes associated with colonization time or host B-cell status. (E) Changes in protist metabolism during colonization. The dot plot represents enrichment of *Tmu* EC terms in KEGG-defined pathways, within sets of genes DE over time or based on host B-cell status. Colored dots represent significantly enriched pathways. Gray dots are ns. Significance was evaluated using Fisher’s exact tests and dot sizes represent odds ratios. The bar chart to the right shows total ECs detected in the experiment. (F) Normalized expression of BspA-like genes showing interaction between time and host B-cell status, and (G) DE BspA-like gene and adhesins homologous to *T. vaginalis* virulence factors. Differences were evaluated using the Wilcoxon rank-sum test and adjusted for multiple testing using the Benjamini–Hochberg approach. ^*^P < .05, ^*^^*^P < .01, ^*^^*^^*^P < .001, ns nonsignificant.

To monitor *Tmu* gene expression during colonization, we generated a draft assembly of the protist genome. Assembly statistics are available in Supplementary Table S1. Functional annotation predicts 26 723 genes, with 999 unique GO terms, 417 enzymes, and 1932 Pfam domains (Supplementary Data 5). We also identified a second distinct rRNA locus with 97% sequence identity to *Tmu* at lower abundance (19% of rRNA reads; Fig. 3A), confirmed as a novel species of the Tritrichomonadidae order through phylogenetic comparison (Fig. 3B).

We mapped 157 million metatranscriptomic reads to the protist, of which 20% mapped to coding regions (Supplementary Data 3). *Tmu* gene expression changed during colonization in both WT and *muMt*^−/−^ hosts (666 and 627 DE genes, respectively), with a host B-cell-modulating effect apparent at day 28 (Fig. 3C and D). Consistent with growth, expression of enzymes involved in energy production (e.g. the tricarboxylic acid cycle and glycolysis) and carbohydrate metabolism (e.g. galactose, and starch and sucrose) increased over time (Fig. 3E). We probed genes with potential roles in establishing colonization: adhesins, BspA proteins, lectins, and cysteine proteases (Supplementary Data 6, 7), protein families previously implicated in facilitating host interactions and parasite infection [23, 58–61]. Adhesins AP33 and AP120, and BspA625, homologs of which mediate virulence and host cytoadherence in *T. vaginalis* [23, 58], were upregulated at day 28 in WT mice. These genes exhibited increased expression already at day 2 in *muMt*^−/−^ mice, consistent with accelerated protist expansion in the absence of host B cells (Fig. 3F). The expression of four additional BspA proteins with predicted extracellular immunogenic regions changed in B-cell-dependent manners, upregulated only in WT mice (TMU_00001180, TMU_00003472, TMU_00005602, TMU_00010916; Fig. 3G, Supplementary Data 8). The remaining genes showed varied changes during colonization and/or in the presence of B cells (Supplementary Fig. S5B), suggesting dynamic metabolic and accessory functions as the protist adapts to the host environment. Future research is set to validate the role of these proteins.

### Single-cell profiling reveals *Tmu* life cycle stages

**Figure 4:**
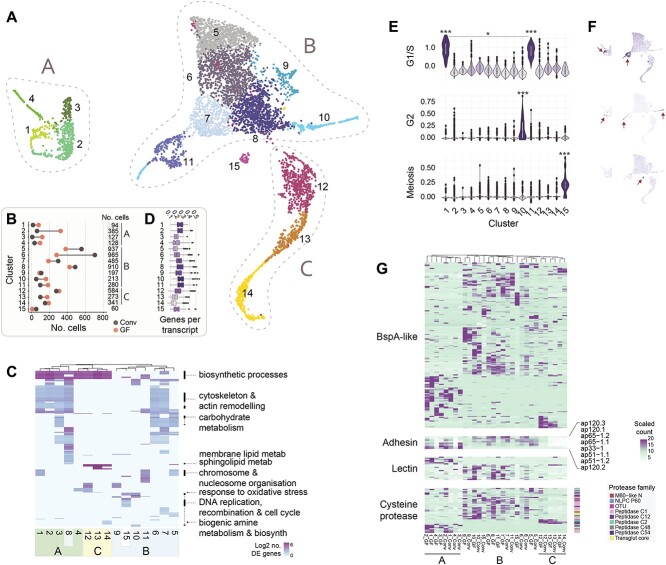
Single-cell transcriptional profiling of caecal *Tmu* populations. (A) Protist subpopulations isolated from colonized GF and conventionalized mouse caeca. Each point represents one of 6000 protists cells, clustered by gene expression profiles using UMAP dimensionality reduction and colored by cluster designation (3000 cells from each of two hosts). Groups of clusters, designated superclusters A, B, or C, are indicated. (B) Numbers of cells assigned to each cluster by host are shown. Total cluster sizes are shown on the right. (C) Heatmap of GO term enrichment within *Tmu* clusters. Rows represent significantly enriched GO terms in one or more clusters (columns). Cells are colored and clustered by (log-transformed) numbers of upregulated transcripts per GO term. (D) Transcriptional complexity of protist clusters, defined as numbers of genes per transcript. Boxplots indicate medians and interquartile ranges. (E) Violin plots and (F) feature plots depicting G1/S, G2, and meiosis scores in cells across each *Tmu* cluster. Scores were determined based on the average expression of predefined gene sets relative to randomly chosen control genes. Significance was evaluated using the two-sided Wilcoxon rank-sum test, ^*^P < .05, ^*^^*^P < .01, ^*^^*^^*^P < .001. Asterisks above clusters indicate significantly higher score relative to all other clusters. Lines denote specific comparisons between two clusters. (G) Heatmap showing expression of (*top* to *bottom*) BspA-like proteins, putative adhesin proteins, lectins, and cysteine proteases across protist clusters. Colors to the right of cysteine proteases indicate the protein family (Pfam).

The trichomonad life cycle features a variety of cell states (e.g. trophozoite, amoeboid, pseudocyst). To characterize these states in *Tmu*, and whether they are influenced by resident bacteria and host B cells, we carried out single-cell transcriptional profiling in protists isolated from a conventionalized and GF mouse, as GF mice are also known to possess an immature B-cell compartment (Supplementary Table S2) [62]. Clustering of transcriptional profiles from 6000 protists (3000 per host) revealed 15 distinct populations, assigned to three “superclusters” (Fig. 4A). Clusters 1–4 (designated supercluster A) and clusters 10 and 11 were enriched in protists from the GF mouse, whereas clusters 5–7 and 15 were composed primarily of conventionalized mouse protists (Fig. 4B).

GO term enrichment suggested the presence of actively metabolizing cells, protists at various stages of cell cycle, and those undergoing pseudocyst formation (Fig. 4C). Clusters 1–3 and 5–8, for example, were enriched in cytoskeleton and actin remodeling, carbohydrate metabolism, and biosynthetic processes and may represent actively feeding and growing trophozoites. Cluster 8 is enriched in sphingolipid metabolism, previously documented to regulate *Giardia* encystation [63]. Since cluster 14 is enriched in biogenic amine metabolism, a pathway associated with encystation in *Entamoeba invadens*, and exhibits lowest transcriptional complexity (Fig. 4D), we suggest these cells represent *Tmu* pseudocyst forms [64–66]. Furthermore, the upregulation of oxidative stress response in neighboring clusters 12 and 13 suggests the encystation transcriptional program may be triggered through stress-induced pathways.

Enrichment of chromosome reorganization, DNA replication, and cell cycle checkpoint in clusters 1, 5, 10, and 11 identified protists undergoing cell cycle. Although cluster 15 shared these cell cycle terms, it was uniquely enriched in DNA recombination, indicative of sexual or parasexual replication. To investigate further, we calculated enrichment scores for the expression of genes specific to G1/S and G2 phases of the cell cycle and meiosis (Supplementary Data 6 and 7) [22, 67]. G1/S scores were highest in clusters 1 and 11, G2 in cluster 10, and cluster 15 scored highest for meiosis (Fig. 4E and F; Supplementary Fig. S6A and B). In addition to the meiosis-specific genes *dmc1*, *hop2A*, and *mnd1*, required genes *rad1*, *mre11*, *smc2*, *smc3*, and *smc5* were also significantly upregulated in cluster 15 (Supplementary Fig. S6C), consistent with sexual or parasexual replication. Of the 60 cells, which compose this cluster, only eight were from the GF mouse. Although the differences in conventionalized versus GF protist distributions in clusters may implicate the microbiome in modulating the protist life cycle (Fig. 4B), we note that despite extensive protist purification and antibiotic treatment, we detected bacterial DNA in the GF mouse, thus limiting our conclusions.

### Protist cell states express distinct virulence factors

We investigated whether protist cell states differ in their expression of genes associated with colonization and/or virulence: BspA proteins, adhesins, lectins, and cysteine proteases (Fig. 4G, Supplementary Data 9). Many genes exhibited cluster and/or host specificity, with profiles broadly defined by the three major superclusters. Notably, cells in cluster 15 (the putative sexual stage) expressed distinct BspA genes in GF and conventionalized hosts, whereas putative encysting stages (clusters 12–14) shared similar expression patterns in both mice. Adhesins, shown in *T. vaginalis* to mediate binding to host epithelia [23], were absent from pseudocyst-associated clusters 13 and 14, supporting their role in colonization. Lectins were also depleted in clusters 12–14, as well as in cluster 15. Of the four cysteine proteases expressed in putative pseudocysts, two belong to the C54 peptidase family that has been implicated in cell starvation and differentiation [68, 69]. This dynamic arsenal of cell state and host-specific virulence factors, potentially modulated by resident microbiota, may mediate protist colonization and transmission.

### Distinct *Tmu* cell states are detected *in vivo*

**Figure 5:**
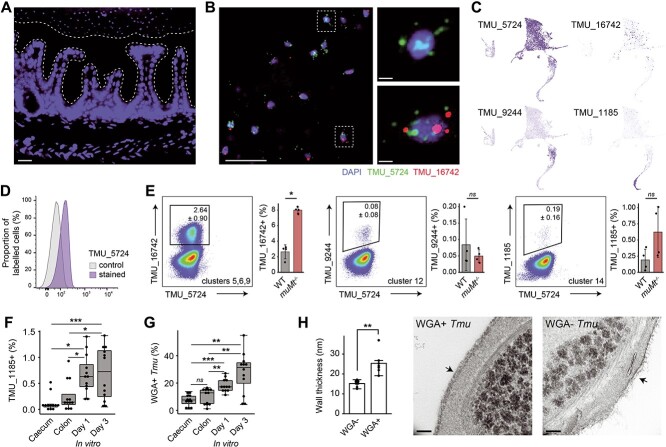
*Tmu* subpopulations are detected *in situ*. (A) Immunofluorescence micrograph of mouse caecal tissue after colonization with *Tmu* for 21 days. Nuclear staining (blue) shows host and protist DNA, and white dotted lines denote the mucus layer. Scale bar = 10 μ*m*. (B) RNAscope immunofluorescence of *Tmu* transcripts TMU_00005724 (green) and TMU_00016742 (red) on sections of caecal tissue. Nuclear DNA is stained blue. Scale bars, left image = 10 *μ*m; right images = 1 *μ*m. (C) Feature plots showing the expression of cluster-specific genes. (D) FISH-flow analysis of freshly isolated *Tmu* at 21 days post colonization. Histogram shows control unstained cells (gray) and cells stained with fluorescent probes for TMU_00005724 (purple). (E) FISH-flow analysis of *Tmu* isolated 28 days post colonization from WT or *muMt^−/−^* mice. Numbers adjacent to gates represent the average percentage ± standard deviation of probe-expressing *Tmu*. Data shown is the representative of four independent experiments and adjacent plots show results from all experiments. Significance was tested using the Wilcoxon rank-sum test. (F) Percentages of TMU_00001185 probe-positive or (G) WGA-FITC-stained protists freshly isolated from WT mouse caeca and colons, or cultured *in vitro* for 1 and 3 days. *n* = 3 animals or culture plates per group from four independent experiments. Significance was tested using one-way ANOVA and adjusted for multiple comparisons using Tukey’s test. (H) Cell wall thickness of FACS-sorted protists hybridized or not to WGA-FITC. Cells were imaged using TEM and wall thicknesses were measured for all cells [5–10] across seven view fields per group. Scale bars = 100 nm. Bars are means and whiskers show interquartile ranges. ^*^P < .05, ^*^^*^P < .01, ^*^^*^^*^P < .001, ns not significant.

We validated the presence of distinct *Tmu* populations in the mouse intestine through *in situ* fluorescent labeling of cluster-specific transcripts. Microscopic imaging of caecal sections from WT mice revealed protists as large, nucleated cells restricted to the gut lumen (Fig. 5A). Protists expressing one or more cell-state-specific gene could be visibly identified in the caecum (e.g. TMU_00005724, specific to cells of supercluster B, and/or TMU_00016742, expressed in a subset of B; Fig. 5B and C). The expression of TMU_00005724 was detected in all caecal protists (Fig. 5D). Consistent with cluster-specific profiles, expression of TMU_00016742 (clusters 5, 6, and 9) exhibited higher overlap with TMU_00005724 than did TMU_00009244 (cluster 12) and TMU_00001185 (cluster 14) (Fig. 5C and E). *muMt^−/−^* mice harbored more TMU_00016742-expressing protists, suggesting B-cell influence on the *Tmu* transcriptional program (Fig. 5E). Since TMU_00001185 was unique to cells of cluster 14 (predicted pseudocysts) and pseudocyst formation occurs during host egress, we tested its expression in protists freshly isolated from the caecum, colon, and for up to 3 days of *in vitro* culturing (Fig. 5F). As expected, caecal isolates contained fewest TMU_00001185-expressing cells and their proportions increased during *in vitro* culturing. Supporting their identity as pseudocysts, the same pattern was observed for presence of chitin, a critical component of cysts and pseudocysts, stained with fluorophore-labeled WGA (Fig. 5G, Supplementary Fig. S7) [66, 70, 71]. WGA-labeled protists furthermore had thicker cell walls, consistent with cyst-like structures (Fig. 5H). The *in situ* data confirm the presence of transcriptionally distinct protist populations in the caecum, and illustrate a powerful method to track *Tmu* dynamics during colonization.

### *Tmu* and gut bacteria compete for nutrients

**Figure 6:**
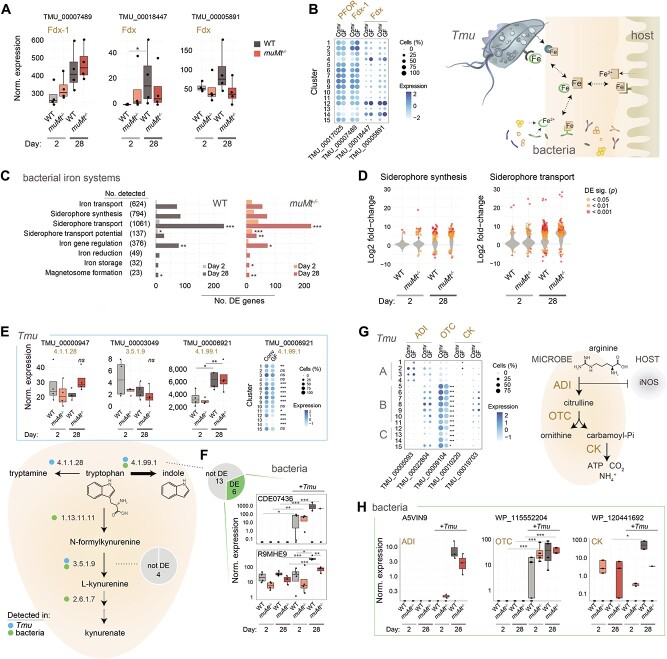
*Tmu* and resident bacteria compete for resources in the gut lumen. Shown are changes in expression of bacterial and *Tmu* genes associated with iron, A–D, generation of tryptophan catabolites, E and F, and the arginine dihydrolase pathway, G and H. (A) Expression of putative *Tmu* Fdx genes in metatranscriptomic data at days 2 and 28. (B) PFOR and Fdx gene expression among protist subpopulations isolated from GF and conventionalized mice. Color intensity correlates with average expression across cells and dot sizes represent percentages of cells in each cluster with detected expression. (C) Enrichment of iron-related gene expression in bacteria, in the context of *Tmu* colonization. Shown are numbers of DE genes per iron-related gene family in *Tmu*-colonized versus naïve mice at days 2 and 28. Total numbers of genes identified in each family in the metatranscriptomic data are indicated in brackets. Significance of enrichment was tested using the hypergeometric test. (D) Median log2 fold-change of bacterial siderophore transport and synthesis gene expression between colonized and naïve mice. Significantly DE genes are shown as colored points. (E) Expression of *Tmu* and (F) the most highly expressed bacterial (read count > 100) enzymes with predicted functions in the production of tryptophan metabolites. Dot plots in E show gene expression among protist subpopulations as above. Pie charts in F represent the total numbers of tested and DE bacterial enzymes. Blue and green dots in the graphic show enzyme activities identified in *Tmu* and/or bacteria, respectively. (G) Expression of *Tmu* enzymes associated with the arginine dihydrolase pathway among protist subpopulations isolated from GF and conventionalized mice, as above. (H) Expression of bacterial enzymes associated with the arginine dihydrolase pathway. Shown are the only ADI present in the rarefied gene count matrix and the two DE OTC and CK genes. Gene expression was evaluated using DESeq2 for metatranscriptomics data or Seurat for scRNA-Seq data. All expression values are derived from normalized gene counts. Boxplots and violin plots show medians and interquartile ranges. *P* values were corrected for multiple testing using the Benjamini–Hochberg approach. ^*^P < .05, ^*^^*^P < .01, ^*^^*^^*^P < .001.

As *Tmu* colonizes the gut, it must compete with resident microbiota and the host for nutrients. Since trichomonads require high concentrations of iron for growth [72, 73], we hypothesized that its consumption by *Tmu* would exert pressure on bacterial iron acquisition and storage systems. Metatranscriptomic data confirmed increasing protist iron consumption through the upregulation of ferredoxin (Fdx)-1 (TMU_00007489), an enzyme required for energy production, and adhesin TMU_00017025 with predicted pyruvate:Fdx oxidoreductase activity (Figs 6A and 3F) [74, 75]. Both enzymes were widely expressed across protist cell states, but reduced in clusters 13 and 14 (Fig. 6B). The upregulation, instead of Fdx TMU_00018447 and TMU_00005891 in clusters 12 and 14, may be specific to encysting forms and potentially involved in mediating stress response. Similar expression in conventionalized and GF mice suggests that *Tmu* is agnostic in terms of iron acquisition to the resident microbiome. In response to *Tmu*, expression of bacterial iron acquisition and storage systems increased, including siderophore synthesis and transport genes (Fig. 6C and D), predominantly in *Helicobacter*, *Bacteroides*, *Parabacteroides*, and *Mucispirillum schaedleri* (Supplementary Fig. S8B). Host B-cell status minimally impacted these systems, suggesting more direct competition between *Tmu* and resident microbiota (Supplementary Fig. S8C).

We also probed genes associated with tryptophan and arginine metabolism, amino acids implicated in host:microbiome interactions [76, 77]. Protist tryptophanase TMU_00006921 (EC 4.1.99.1) was upregulated at day 28, whereas enzymes in competing pathways (conversion of tryptophan to kynurenate and tryptamine) exhibited minimal expression and no temporal shift, suggesting that *Tmu* drives production of indole (Fig. 6E). The two most highly expressed bacterial tryptophanases were upregulated in *Tmu* colonized-mice suggesting further protist-induced bacterial production of indole (Fig. 6F). Consistent with this, scRNA-Seq data showed increased expression of *Tmu* tryptophanase in the conventionalized mouse and reduction in putative pseudocyst clusters suggesting reduced dependence on host mucosal homeostasis (Fig. 6E).

Arginine is an important source of energy for both trichomonads and bacteria. Its depletion by microbes through the arginine dihydrolase pathway has an additional immunomodulatory role, limiting host production of antimicrobial NO. [76, 77]. None of the three *Tmu* enzymes in the associated pathway: arginine deiminase (ADI), ornithine transcarbamylase (OTC), or carbamate kinase (CK), were significantly associated with either colonization time or host B-cell status, suggesting continual arginine consumption (Supplementary Fig. S5D). We did note selective expression across protist cell states (Fig. 6G). Conversely, protist colonization induced the expression of several bacterial arginine dihydrolase genes, including Lactobacillales ADI and *Helicobacter* OTC (Fig. 6H).

The dynamics of the acquisition and metabolism of these key gut nutrients suggests competitive and cooperative relationships between the protist and the resident microbiota.

## Discussion

Previous work has revealed that protists mediate antagonistic and mutualistic interactions with intestinal bacteria with consequences for the host, primarily through studies of microbial composition [6, 7, 13, 78, 79]. Here we demonstrated interactions between *Tmu* and resident microbiota at a functional level in a healthy and immunodeficient host, and identified genes predicted to facilitate protist colonization. We showed that *Tmu* induces longitudinal shifts in bacterial composition and increases abundance and activity of *Helicobacter* spp., consistent with previous trichomonad studies [15, 80]. Although previous studies have shown natural microbiome variation in mice over their lifespan [81], our metatranscriptomic data in particular suggest little change over the 28 days of the experiment. As mice were co-housed prior to protist exposure to normalize their microbiomes, the similarity of 16S rRNA compositions in day 28 control mice to day 0 preinfection samples, relative to the large *Tmu*-driven shift in the colonized mice, further supports limited variation during this period. The shift from Firmicutes to Bacteroidota, Campilobacterota and Proteobacteria in colonized mice suggests environmental pressure favoring Gram negative taxa, which may contribute to the intestinal immune activation observed during *Tmu* engraftment through immune-modulating surface LPS [6, 82]. The earlier occurrence of these changes in B-cell-deficient mice suggests a failure to control commensal bacteria that might otherwise be mediated through *Tmu*-induced IgA reactivity [17, 83, 84]. We speculate this reactivity might serve to limit adverse responses by the bacteria as the protist attempts to colonize. Similarly, the accelerated expansion of *Tmu* and upregulation of its adhesins and TMU_00015121, a homolog of the antigenic *T. vaginalis* BspA625 protein, signal changes in protist immunogenicity and suggest that B-cell surveillance plays a critical role during early protist infection [58, 85]. In addition to humoral immunity, it is important to note that adaptive immunity also contributes to interkingdom interactions [86, 87]. For example *Tmu* colonization activates Th1 and Th17 cells, where the Th1 response is associated with resistance to *Salmonella* challenge, and both the Th17 response and protist expansion are increased in the absence of resident bacteria [6].

Our single-cell profiling demonstrated distinct stages of the *Tmu* life cycle. Cell state-specific expression patterns of protein families implicated in host cell binding or parasite virulence suggest *Tmu* alters its host adhesive properties and antigenicity as it transitions through its life cycle [23, 58, 60, 61]. As trichomonads exist in one of three forms—actively growing trophozoites, host-adherent amoeboid cells, and environmentally resistant pseudocysts—the capture of actively metabolizing *Tmu* in the lumen and cells exhibiting reduced activity (with little to no expression of adhesins), thicker cell walls, and stress-activated pathways suggests we captured the trophozoite and pseudocyst states [65, 66, 88–90]. We validated the pseudocyst stage through TEM and staining of chitin and cluster-specific transcripts in mouse intestinal contents and *in vitro*. A similar transcript-labeling approach could be used to capture the *Tmu* amoeboid state after exposure to host epithelia, a clinically relevant cell type associated with pathogenicity [88, 89]. One possibility is that we have already captured this state in supercluster A, which expresses different suites of virulence factors and enzymes involved in arginine metabolism. The majority of this population is composed of protists from the GF mouse, known to have a more penetrable intestinal mucus [91]. Supporting the ability of *Tmu* to penetrate the intestinal mucus layer, we detected a large repertoire of expressed cysteine proteases and enzymes of the N-glycan degradation pathway capable of degrading O-glycans, the dominant glycans of mucins [92, 93].

The discovery of meiosis-specific genes is particularly intriguing as it suggests the presence of sexual replication, with implications for host adaptation and immune evasion. Sexual recombination has previously been hypothesized in *T. vaginalis* and *Giardia duodenalis* [22, 94–97]. Our data may furthermore implicate the microbiome in promoting meiosis, as noted by the low proportion of meiotic cells in the GF mouse. As we detected bacterial DNA in the GF mouse, these differences may arise from timing of microbiome exposure. The conventionalized mouse was colonized with commensal bacteria 4 weeks prior to *Tmu* infection, which would have affected intestinal morphology and immune cell differentiation. Additional experiments are needed to confirm the impact of the microbiome on *Tmu*, however, to date attempts at axenic culturing of *Tmu* have proven challenging [98].

To successfully colonize, *Tmu* must compete with resident bacteria for nutrients. In particular, parasitic trichomonads are known to require high quantities of iron to sustain growth, whereas low iron conditions hinder their adhesion to host epithelia [73, 99]. To ward off invading pathogens, hosts restrict access to luminal iron through sequestration by proteins such as lactoferrin [72, 73, 100]. The upregulation of bacterial iron acquisition in response to *Tmu* has the potential, therefore, to regulate protist growth and virulence, an effect which may be lost in a context of surplus iron provided by nutritional supplements [5]. We also noted synergistic expression of bacterial and protist genes which metabolize tryptophan to indole. Microbial indoles, produced also by *Tritrichomonas foetus* and *T. vaginalis*, are known to impact host immunity and barrier function [101–103]. By furthermore promoting production of indole at the expense of alternative products such as neuroactive kynurenine, our findings propose that protists, like the resident microbiome, may modulate the gut–brain access [104, 105]. Metabolomics will be crucial to validate these findings. In this study, we provide a detailed blueprint of gut ecosystem changes induced by protist colonization, which will contribute to future mechanistic studies, and confirm *Tmu* as a powerful modulator of the murine gut microbiome.

## Supplementary Material

Supplementary_Data_1

Supplementary_Data_2

Supplementary_Data_3

Supplementary_Data_4

Supplementary_Data_5

Supplementary_Data_6

Supplementary_Data_7

Supplementary_Data_8

Supplementary_Data_9

Supplementary_Information

## Data Availability

Sequence data generated in this study have been deposited to the NCBI Sequence Read Archive under the BioProject identifiers PRJNA913581 and PRJNA914770.
