# Supplementary material for: Commensal protist Tritrichomonas musculus exhibits a dynamic life cycle that induces extensive remodeling of the gut microbiota
Source: ISME J. 2024 Feb 5;18(1):wrae023. doi: 10.1093/ismejo/wrae023 (PMC10944700; doi:10.1093/ismejo/wrae023)
Supplement: Supplementary_Information [file supplementary_information.pdf]

Supplementary Information:

Commensal protist *Tritrichomonas musculus* exhibits a dynamic life cycle that induces extensive remodeling of the gut microbiota

The supplementary information file contains:

Supplementary Methods

Supplementary Tables 1-2

Supplementary Figures 1-8

Supplementary Methods

Genome sequencing and annotation

Genomic DNA was extracted from 100 million sorted protists using the MagAttract HMW DNA Kit (QIAGEN, Hilden, Germany). Sequencing libraries were prepared and sequenced using Sequel technology (PacBio, Menlo Park, CA, USA) on two SMRT cells at the McMaster University Farncombe Metagenomics Facility (Hamilton, Canada). Reads were error corrected using CANU v1.8[1] and assembled into contigs with Flye v.2.4.1[2]. The contigs were subsequently polished with eight million 300 bp paired-end reads generated on a MiSeq System (Illumina, San Diego, CA, USA), sequenced at the National Institute of Allergy and Infectious Diseases (Bethesda, Maryland), using BWA[3] and Pilon v.1.23[4]. Annotation was carried out with Maker v2.31[5] as follows. Repetitive regions were identified *de novo* and masked using RepeatModeler v.1.0.11[6] and RepeatMasker v.4.0.7[7]. Transfer RNA genes were predicted with tRNAscan-SE v2.0.6[8]. Gene models were predicted *ab initio* using two rounds of training with SNAP v.2013-11-29[9], followed by Augustus v.3.3.1[10] with BUSCO v.2.0.1[11]. Reference sequences included related trichomonad *T. foetus* expressed sequence tag (EST) library (CX159216.1), *T. foetus* strain K (3AUP000179807) and *Trichomonas vaginalis* G3 (3AUP000001542) proteomes, and *Pentatrichomonas hominis* and *Dientamoeba fragilis* protein sequences available in the NCBI nr database (accessed August 21, 2019). Functional annotation was performed with InterProScan v.5.30-69.0[12], the HmmerWeb v.2.41.2[13] hmmscan algorithm (E-value ≤1e-05) and Architect[14] (confidence ≥0.5). Genes encoding adhesins, meiosis, and cell cycle-related proteins were identified based on sequence homology with *T. vaginalis* proteins[15,16] retrieved from the TrichDB database[17] using BLAST[18] (E-value 1e-5, 30% sequence identity and 40% query coverage cut-offs). The genome assembly is available at: https://github.com/ParkinsonLab/Tritrichomonas-murine-microbiome-interactions/.

Phylogenetic analysis

Accessions to parabasalid ribosomal ITS sequences used in the analysis of phylogeny.

Organism	Genbank ID
Monocercomonas colubrorum	AY319266
Trichomonas gallinae	JQ755289
Trichomonas tenax	HM579936
Trichomonas vaginalis	AY871046
Pentatrichomonas hominis	AY349187
Honigbergiella sp.	AY319274
Tritrichomonas musculus	KX000922
Tritrichomonas rainier	MH370486
Tritrichomonas muris	AY886843
Tritrichomonas foetus	KX425890
Tritrichomonas suis	U85967
Tritrichomonas sp. isolate GZH	MF375342
Histomonas meleagridis	HM229782
Dientamoeba fragilis	DQ233449
Trichomitus sp.	KP012660
Trichomitus batrachorum	AY349193

scRNA-Seq

Single cell profiling was carried out for protists isolated from a GF and conventionalized mouse four weeks post colonization. Protists were purified from caecal contents and immediately transferred on ice to the Princess Margaret Genomics Centre (Toronto, Canada) for STAMP library preparation using Drop-seq technology, and sequenced on a NextSeq 500 System (Illumina, San Diego, CA, USA)[19]. Reads were processed using Drop-seq Tools v.1.13 and aligned to the protist genome assembly using STAR v. 2.5.3a[20,21]. Three thousand protists per mouse (minimum 200 genes, 500 transcripts) were analyzed using Seurat v4[22,23]. Cells were grouped using graph-based clustering (0.8 resolution, 18 principal components) and visualized via UMAP[24]. Differentially expressed (DE) genes were identified using the FindAllMarkers function, and functional enrichments were determined based on overrepresentation of pathway enzymes as defined by KEGG using the hypergeometric test or GO terms using the topGO package and the Fisher's Exact test[25,26]. Enrichments of custom-defined gene sets (meiosis, G1/S, and G2 phase genes) were scored with the AddModuleScore function and evaluated using two-sided Wilcoxon rank-sum tests. Benjamini-Hochberg correction was applied for multiple testing[27]. Heatmaps were generated using pheatmap 1.0.12 and Ward.D2 clustering[28].

qPCR

DNA primers used for quantitative PCR of bacterial taxa[29]

Target	primer sequence (5'-3')
**Bacterial taxa**	
Firmicutes_934F	GGAGYATGTGGTTTAATTCGAAGCA
Firmicutes_1060R	AGCTGACGACAACCATGCAC
Actinobacteriota_920F	TACGGCC GCAAGGCTA
Actinobacteriota_1200R	TCRTCCCCACCTTCCTCCG
Gammaproteobacteria_1080F	TCGTCAGCTCGTGTYGTGA
Gammaproteobacteria_1202R	CGTAAGGGCCATGATG
Bacteroidota_798F	CRAACAGGATTAGATACCCT
Bacteroidota_967R	GGTAAGGTTCCTCGCGTAT

RNAScope

RNAScope was performed as per the RNAScope Multiplex Fluorescent Reagent Kit v2 (Advanced Cell Diagnostics, Newark, CA, USA) protocol. Approximately 0.5 cm caecum sections were excised from *Tmu*-colonized mice, placed in 10% neutral buffered formalin, and fixed overnight at room temperature (RT) with gentle agitation. The following day, samples were washed with PBS, placed in 70% ethanol, embedded in paraffin, and sliced to 7 µm sections at the Toronto Centre for Phenogenomics. Paraffin sections were baked at 40°C for 30 min in a HybEZ Oven (Advanced Cell Diagnostics, Newark, CA, USA), and treated with hydrogen peroxide at RT for 10 min. Antigen target retrieval was conducted at 99°C under the 15 min standard procedure. A barrier was created around sections using an ImmEdge pen (Vector Laboratories, Burlingame, CA, USA) and allowed to dry for 15 min. Samples were treated with protease at 40°C for 30 min and stored in saline sodium citrate solution overnight (175.3 g NaCl, 88.2 g sodium citrate, 800 mL ddH_2O, pH 7). TSA Plus Fluorophores Fluorescein and Cyanine 3 were hybridized against protist probes TMU_00005724 and TMU_00016742 respectively, and samples were visualized using a Zeiss AXIO Observer microscope (Carl Zeiss AG, Jena, Germany).

Transmission Electron Microscopy

Protist pellets were prepared using the standard methods for the Embed 812 resin kit (Electron Microscopy Sciences (EMS), Hatfield, PA, USA)[30]. Briefly, samples were fixed with 4% paraformaldehyde, 1% glutaraldehyde in phosphate buffer (PB; 0.1 M, pH 7.2) for 1 hour at room temperature and overnight at 4°C, and washed 3x with PB. They were subjected to a second fixation step with 1% OsO_4 in PB for 1 hour in the dark, and washed 3x with PB for 10 min at RT. Samples were dehydrated in a gradient ethanol series: 30% ethanol for 15 min, 50% ethanol for 20 min, 70% ethanol for 30 min, 90% ethanol for 45 min, and 100% ethanol for 60 min. Dehydrated samples were then infiltrated with the Embed 812 resin kit (EMS) diluted with propylene oxide: 100% propylene oxide for 20 min, 33% (v/v) Embed 812 resin mixture in propylene oxide for 2 hours, 67% (v/v) Embed 812 resin mixture in propylene oxide for 3 hours, 100% Embed 812 resin mixture overnight, and fresh 100% Embed 812 resin mixture for 2 hours. After infiltration, samples in resin were placed in molds and cured at 65°C for 48 hours.

Resin blocks were sectioned to 80 nm thickness with a Reichert Ultracut E microtome (Leica, Wetzlar, Germany), collected on 300 mesh copper grids (EMS), and counter stained for 10 min using saturated 5% uranyl acetate followed by Reynold's lead citrate (EMS). Prepared grids were placed on a filter paper mat in labelled Petri dishes and stored in a desiccator until imaging. The sections were imaged using a Talos L120C transmission electron microscope (Thermo Scientific, Waltham, MA, USA) equipped with a BM-Ceta scientific CMOS camera at an accelerating voltage of 120 KV.

References

1 Koren, S. *et al.* Canu: scalable and accurate long-read assembly via adaptive k-mer weighting and repeat separation. *Genome Res* **27**, 722-736, doi:10.1101/gr.215087.116 (2017).

2 Kolmogorov, M., Yuan, J., Lin, Y. & Pevzner, P. A. Assembly of long, error-prone reads using repeat graphs. *Nat Biotechnol* **37**, 540-546, doi:10.1038/s41587-019-0072-8 (2019).

3 Li, H. Aligning sequence reads, clone sequences and assembly contigs with BWA-MEM. *arXiv:1303.3997v1* (2013).

4 Walker, B. J. *et al.* Pilon: an integrated tool for comprehensive microbial variant detection and genome assembly improvement. *PLoS One* **9**, e112963, doi:10.1371/journal.pone.0112963 (2014).

5 Cantarel, B. L. *et al.* MAKER: an easy-to-use annotation pipeline designed for emerging model organism genomes. *Genome Res* **18**, 188-196, doi:10.1101/gr.6743907 (2008).

6 Smit, A. & Hubley, R. *RepeatModeler Open-1.0* (2008-2015).

7 Smit, A., Hubley, R. & Green, P. *RepeatMasker Open-4.0.*, <http://www.repeatmasker.org> (2013-2015).

8 Chan, P. P. & Lowe, T. M. tRNAscan-SE: Searching for tRNA Genes in Genomic Sequences. *Methods Mol Biol* **1962**, 1-14, doi:10.1007/978-1-4939-9173-0_1 (2019).

9 Korf, I. Gene finding in novel genomes. *BMC Bioinformatics* **5**, 59, doi:10.1186/1471-2105-5-59 (2004).

10 Stanke, M., Schoffmann, O., Morgenstern, B. & Waack, S. Gene prediction in eukaryotes with a generalized hidden Markov model that uses hints from external sources. *BMC Bioinformatics* **7**, 62, doi:10.1186/1471-2105-7-62 (2006).

11 Simao, F. A., Waterhouse, R. M., Ioannidis, P., Kriventseva, E. V. & Zdobnov, E. M. BUSCO: assessing genome assembly and annotation completeness with single-copy orthologs. *Bioinformatics* **31**, 3210-3212, doi:10.1093/bioinformatics/btv351 (2015).

12 Jones, P. *et al.* InterProScan 5: genome-scale protein function classification. *Bioinformatics* **30**, 1236-1240, doi:10.1093/bioinformatics/btu031 (2014).

13 Finn, R. D., Clements, J. & Eddy, S. R. HMMER web server: interactive sequence similarity searching. *Nucleic Acids Res* **39**, W29-37, doi:10.1093/nar/gkr367 (2011).

14 Nursimulu, N., Moses, A. M. & Parkinson, J. Architect: A tool for aiding the reconstruction of high-quality metabolic models through improved enzyme annotation. *PLoS Comput Biol* **18**, e1010452, doi:10.1371/journal.pcbi.1010452 (2022).

15 Malik, S. B., Pightling, A. W., Stefaniak, L. M., Schurko, A. M. & Logsdon, J. M., Jr. An expanded inventory of conserved meiotic genes provides evidence for sex in Trichomonas vaginalis. *PLoS One* **3**, e2879, doi:10.1371/journal.pone.0002879 (2007).

16 Garcia, A. F. & Alderete, J. Characterization of the Trichomonas vaginalis surface-associated AP65 and binding domain interacting with trichomonads and host cells. *BMC Microbiol* **7**, 116, doi:10.1186/1471-2180-7-116 (2007).

17 Aurrecoechea, C. *et al.* GiardiaDB and TrichDB: integrated genomic resources for the eukaryotic protist pathogens Giardia lamblia and Trichomonas vaginalis. *Nucleic Acids Res* **37**, D526-530, doi:10.1093/nar/gkn631 (2009).

18 Altschul, S. F., Gish, W., Miller, W., Myers, E. W. & Lipman, D. J. Basic local alignment search tool. *J Mol Biol* **215**, 403-410, doi:10.1016/S0022-2836(05)80360-2 (1990).

19 Macosko, E. Z. *et al.* Highly Parallel Genome-wide Expression Profiling of Individual Cells Using Nanoliter Droplets. *Cell* **161**, 1202-1214, doi:10.1016/j.cell.2015.05.002 (2015).

20 Nemesh, J. & Wysoker, A. *Drop-seq tools v1.13*, <https://github.com/broadinstitute/Drop-seq/releases> (

21 Dobin, A. *et al.* STAR: ultrafast universal RNA-seq aligner. *Bioinformatics* **29**, 15-21, doi:10.1093/bioinformatics/bts635 (2013).

22 Butler, A., Hoffman, P., Smibert, P., Papalexi, E. & Satija, R. Integrating single-cell transcriptomic data across different conditions, technologies, and species. *Nat Biotechnol* **36**, 411-420, doi:10.1038/nbt.4096 (2018).

23 Hao, Y. *et al.* Integrated analysis of multimodal single-cell data. *Cell* **184**, 3573-3587 e3529, doi:10.1016/j.cell.2021.04.048 (2021).

24 McInnes, L., Healy, J., Saul, N. & Großberger, L. UMAP: Uniform Manifold Approximation and Projection for Dimension Reduction. *Journal of Open Source Software* **3**, 861, doi:https://doi.org/10.21105/joss.00861 (2018).

25 Okuda, S. *et al.* KEGG Atlas mapping for global analysis of metabolic pathways. *Nucleic Acids Res* **36**, W423-426, doi:10.1093/nar/gkn282 (2008).

26 Alexa, A. & Rahnenfuhrer, J. *topGO: Enrichment Analysis for Gene Ontology. R package version 2.42.0.*, <https://github.com/lyijin/topGO_pipeline> (2020).

27 Benjamini, Y. & Hochberg, Y. Controlling the False Discovery Rate: A Practical and Powerful Approach to Multiple Testing. *Journal of the Royal statistical society: series B* **57**, 289-300, doi:https://doi.org/10.1111/j.2517-6161.1995.tb02031.x (1995).

28 Kolde, R. *pheatmap: Pretty Heatmaps. R package version 1.0.12.*, <https://CRAN.R-project.org/package=pheatmap> (2019).

29 Bacchetti De Gregoris, T., Aldred, N., Clare, A. S. & Burgess, J. G. Improvement of phylum- and class-specific primers for real-time PCR quantification of bacterial taxa. *J Microbiol Methods* **86**, 351-356, doi:10.1016/j.mimet.2011.06.010 (2011).

30 Hayat, M. A. *Principles and Techniques of Electron Microscopy*. (Hodder Arnold H& S, 1981).

Supplementary Tables

Supplementary Table 1. Genome assembly characteristics.

**Genome assembly**	
Assembly length (bp)	225,109,209
Scaffolds	1,780
Scaffold N_{50} (bp)	295,434
G+C content (%)	28.7
Portion of genome with repetitive sequences (%)	52.1
**Genes**	
Portion of genome covered by genes (%)	22.3
Genes	26,723
Genes with introns	10,295
CDS	18,405
Exons per mRNA (mean)	2
Mean gene length (bp)	1,878
Mean exon length (bp)	637
Mean intron length (bp)	803
tRNA transcripts (unique)	848

Supplementary Table 2. *Tmu* single cell RNA sequencing statistics.

	GF mouse	Conventionalized mouse
Input reads	232,006,542	224,595,499
Quality filtered reads	231,917,602	224,510,480
Average input read length	59	60
**Read mapping**[a]		
Uniquely mapped reads	162,949,846 (70.3%)	171,330,511 (76.3%)
Reads mapped to no feature	65,802,686 (28.4%)	62,552,460 (27.9%)
Reads ambiguously mapped	1,330,948 (0.6%)	1,191,630 (0.5%)
Average mapped length	60.21	60.58
Mismatch rate per base (%)	2.7%	2.5%
Reads mapped to multiple loci	24,904,419 (10.7%)	21,968,885 (9.8%)
Reads mapped to too many loci	4,119,855 (1.8%)	4,002,291 (1.8%)
Unmapped reads	44,063,337 (19.0%)	31,211,084 (13.9%)
Too short	36,411,064 (15.7%)	23,887,915 (10.6%)
Other	3,525,148 (1.5%)	3,322,755 (1.5%)
Chimeric reads	0 (0%)	0 (0%)
**DropSeq cell statistics**		
Cells selected	3,000	2,999[b]
Reads	69,299,617	70,897,116
Reads per cell (minimum; median; maximum)	3,999; 18,752; 171,812	7; 19,958; 137,383
Transcripts	95,816,212	107,586,421
Transcripts per cell (minimum; median; maximum)	838; 3,776; 28,849	768; 3,140; 20,547
Transcripts mapped to rRNA genes	4163	458
Genes (protein-coding)	20,338	19,436
Genes per cell (minimum; median; maximum)	248; 823; 3,751	201; 589; 1,970

[a]Read mapping percentages were calculated as a proportion of quality filtered reads.

[b]One cell removed due to low gene and transcript detection.

Supplementary Figures

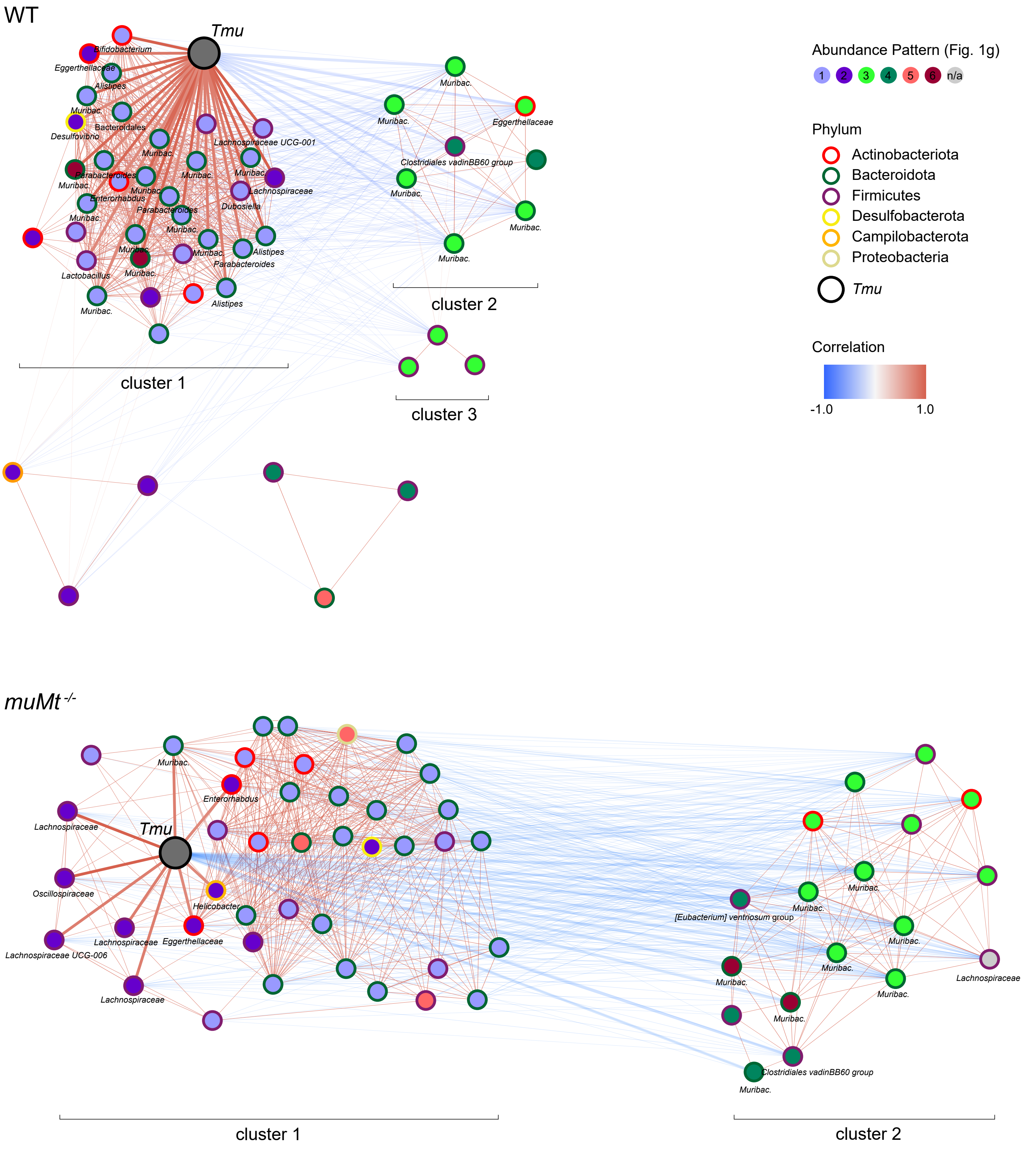

Supplementary Figure 1. Microbial interaction networks depicting interbacterial and *Tmu*-bacterial correlations over time. Nodes represent bacteria or *Tmu*, as indicated, and were clustered using the Markov Cluster Algorithm. Node colours represent the abundance pattern identified in Fig. 1g, and border colours the taxonomic phylum, as detailed in the legend. Edges are positive (red) or negative (blue) correlations based on Kendall τ coefficients (strength of correlation ≥ 0.7). Bacteria directly correlated with *Tmu* are named and linked with thick lines. *Muribac.* = *Muribaculaceae.*

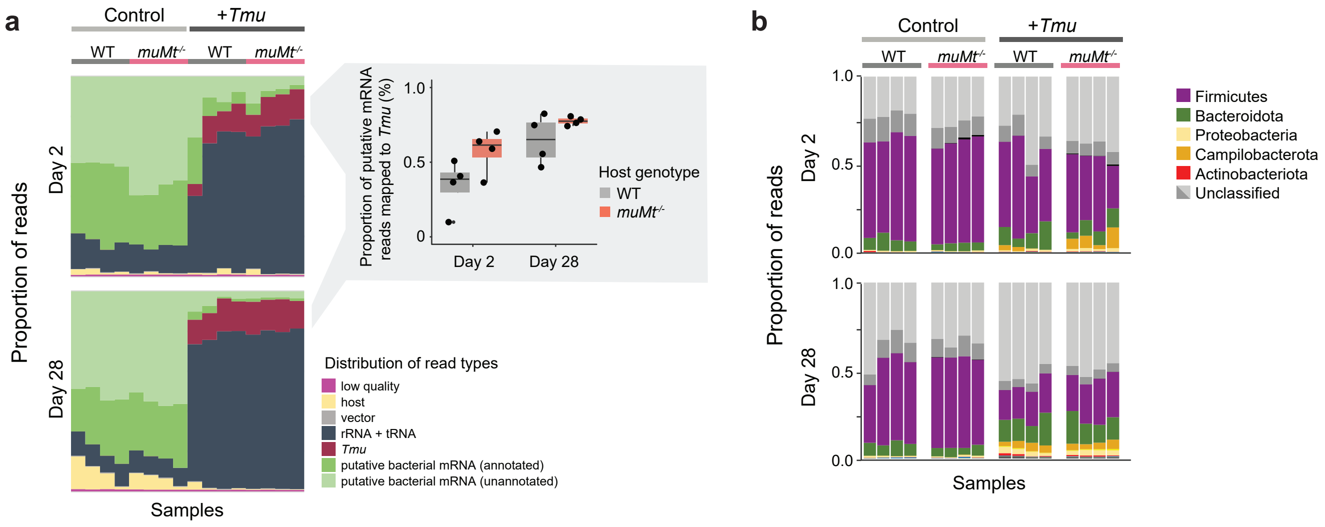

Supplementary Figure 2. Caecal metatranscriptomics of *Tmu*-colonized and naïve (control) WT and *muMt*$^{-/-}$ mice. **a**, Breakdown of caecal RNA reads from filtering and annotation steps. Columns represent samples from individual mice. Outset graph to the right shows percentages of putative mRNA reads mapped to the *Tmu* genome assembly. **b**, Taxonomic classification of putative bacterial transcripts.

Glycolysis/Gluconeogenesis, pentose phosphate and TCA

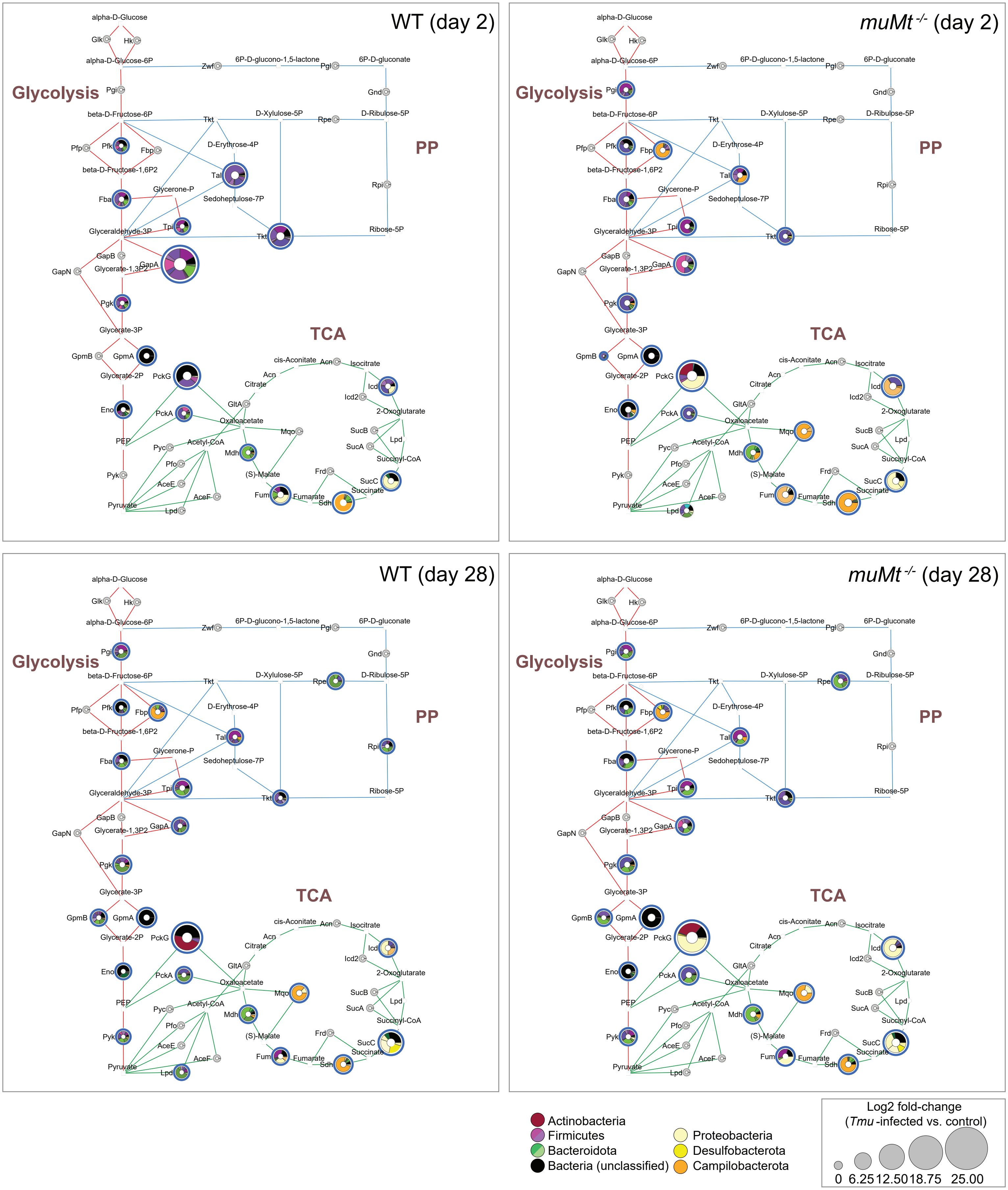

Supplementary Figure 3. Upregulation of bacterial metabolism in response to protist colonization. Depicted are glycolysis/gluconeogenesis (ec00010), tricarboxylic acid (TCA) cycle (ec00020) and the pentose phosphate (PP) (ec00030) pathways in gut microbiota after 2 or 28 days of infection in WT or B cell-deficient ($muMt^{-/-}$) hosts. Genes significantly up- and downregulated ($p < 0.05$ in DESeq2 analysis) are indicated with blue and red borders, respectively. Sizes of nodes represent log2 fold-changes between *Tmu*-colonized and uninfected control mice (n=4 per group). Pie charts depict the phylogenetic source of the gene expression as follows: yellows represent Proteobacteria or Desulfobacterota; orange is Campilobacterota (*Helicobacter*); shades of green are Bacteroidota (dominated by *Bacteroides* and *Parabacteroides*); pinks and purples are Firmicutes (dominated by *Lachnospiraceae* and *Clostridium*); black represents unclassified bacteria.

Valine, leucine and isoleucine biosynthesis (ec00290)

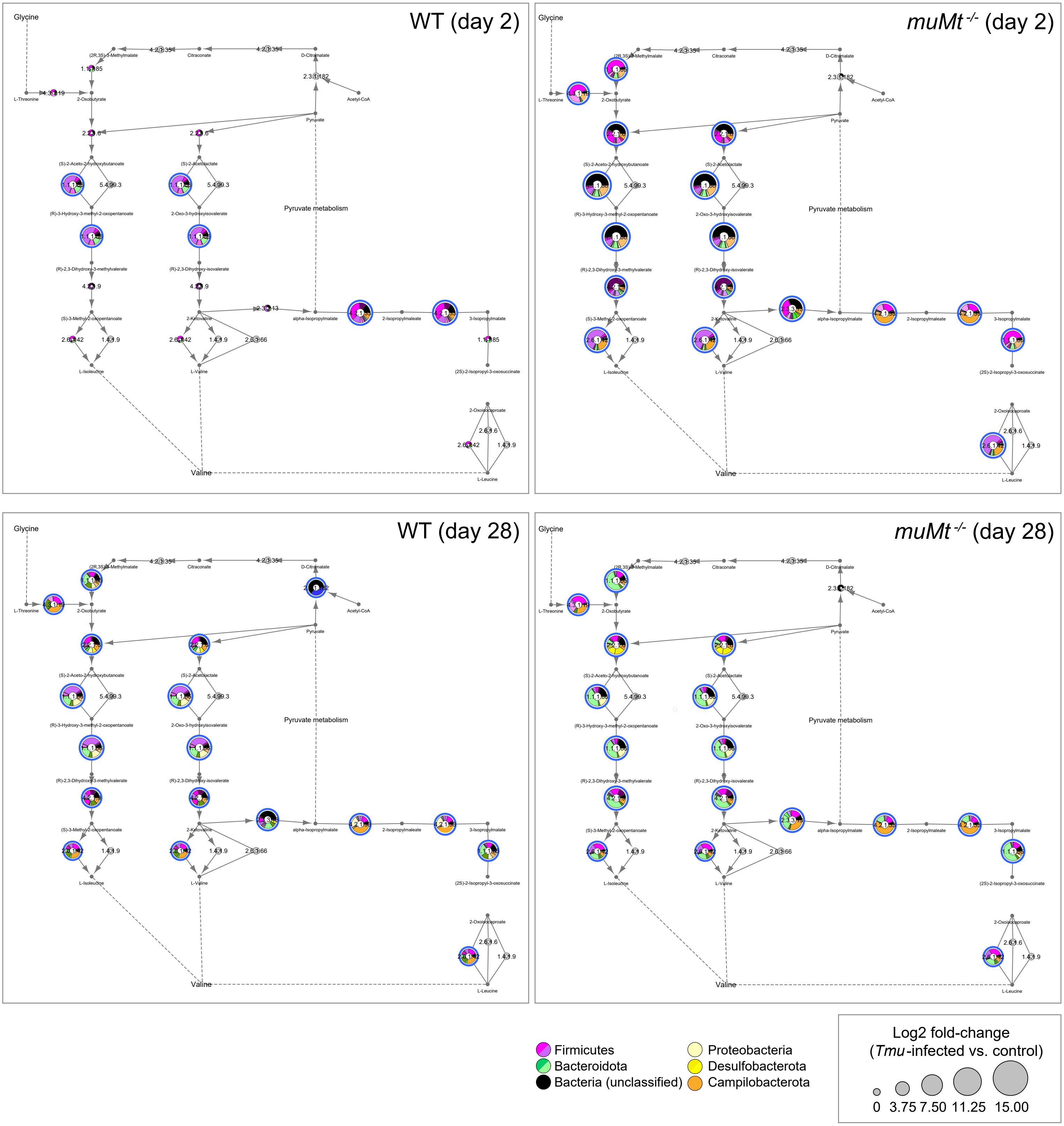

Supplementary Figure 4. Upregulation of Valine-Leucine-Isoleucine biosynthesis in mouse caecal microbiota in response to four weeks of protist colonization. Depicted are the KEGG-based metabolic pathways (ec00290). Genes significantly up- and downregulated (p <0.05 in DESeq2 analysis) are indicated with blue and red borders respectively. Sizes of nodes represent log2 fold-changes between *Tmu*-colonized and uninfected control mice at day 28 of the experiment. Pie charts depict the phylogenetic source of the gene expression as follows: yellows represent Proteobacteria or Desulfobacterota; orange is Campilobacterota (*Helicobacter*); shades of green are Bacteroidota (dominated by *Bacteroides* and *Parabacteroides*); pinks and purples are Firmicutes (dominated by *Lachnospiraceae* and *Clostridium*); black represents unclassified bacteria.

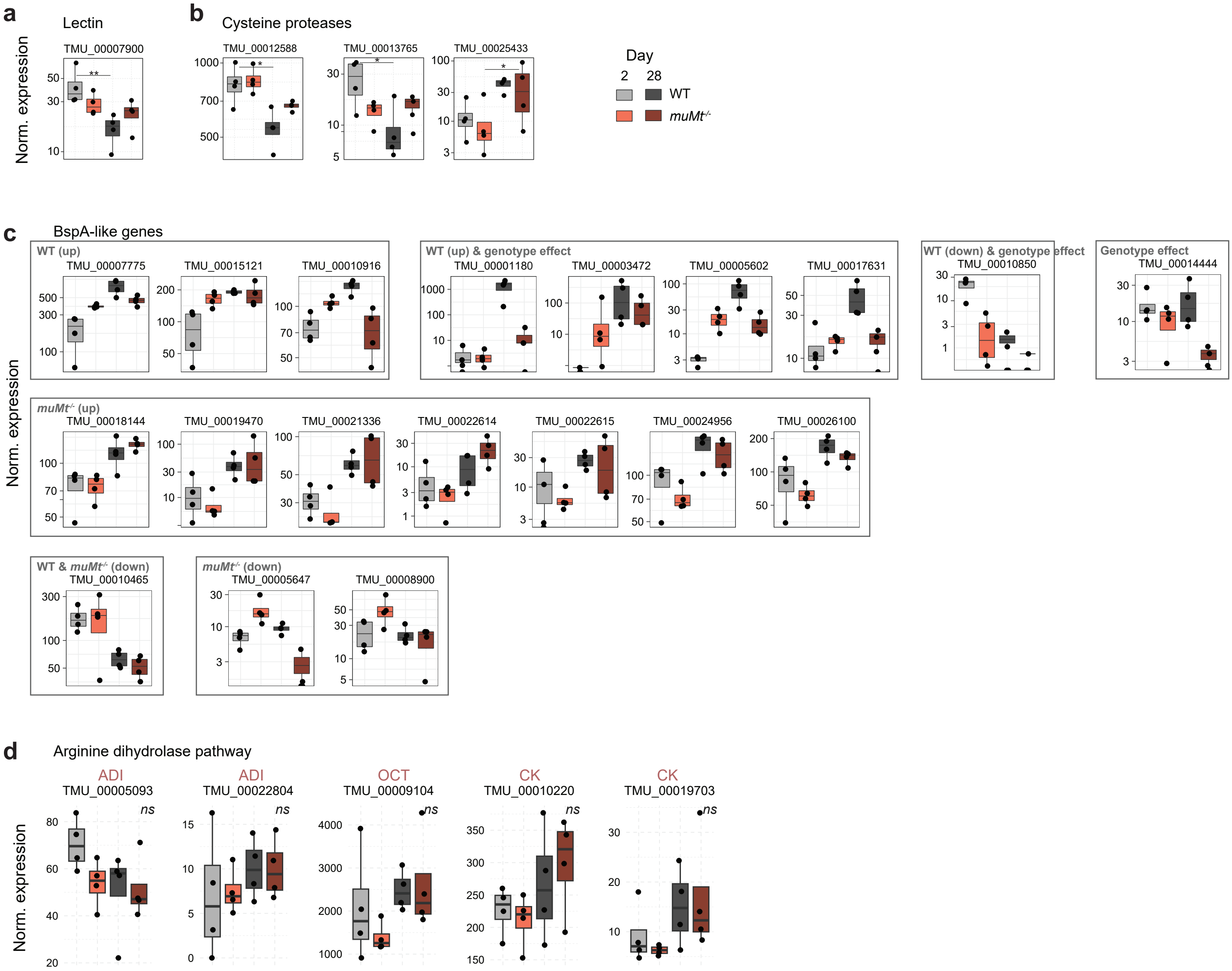

Supplementary Figure 5. Putative virulence-related *Tmu* genes differentially expressed over the course of colonization. **a**, Lectin and **b**, cysteine proteases differentially expressed at 28 compared to 2 days post colonization in WT or *muMt*$^{-/-}$ mice as indicated. **c**, BspA-like genes with significant changes in expression over colonization time and/or which differ between host genotypes. **d**, Expression of genes with predicted activity in the arginine dihyrdolase pathway: arginine deiminase (ADI), ornithine carbamoyltransferase (OCT), and carbamate kinase (CK). Differences in gene expression were tested using DESeq2. **p* <0.05, ** <0.01, *** <0.001, *ns* non-significant

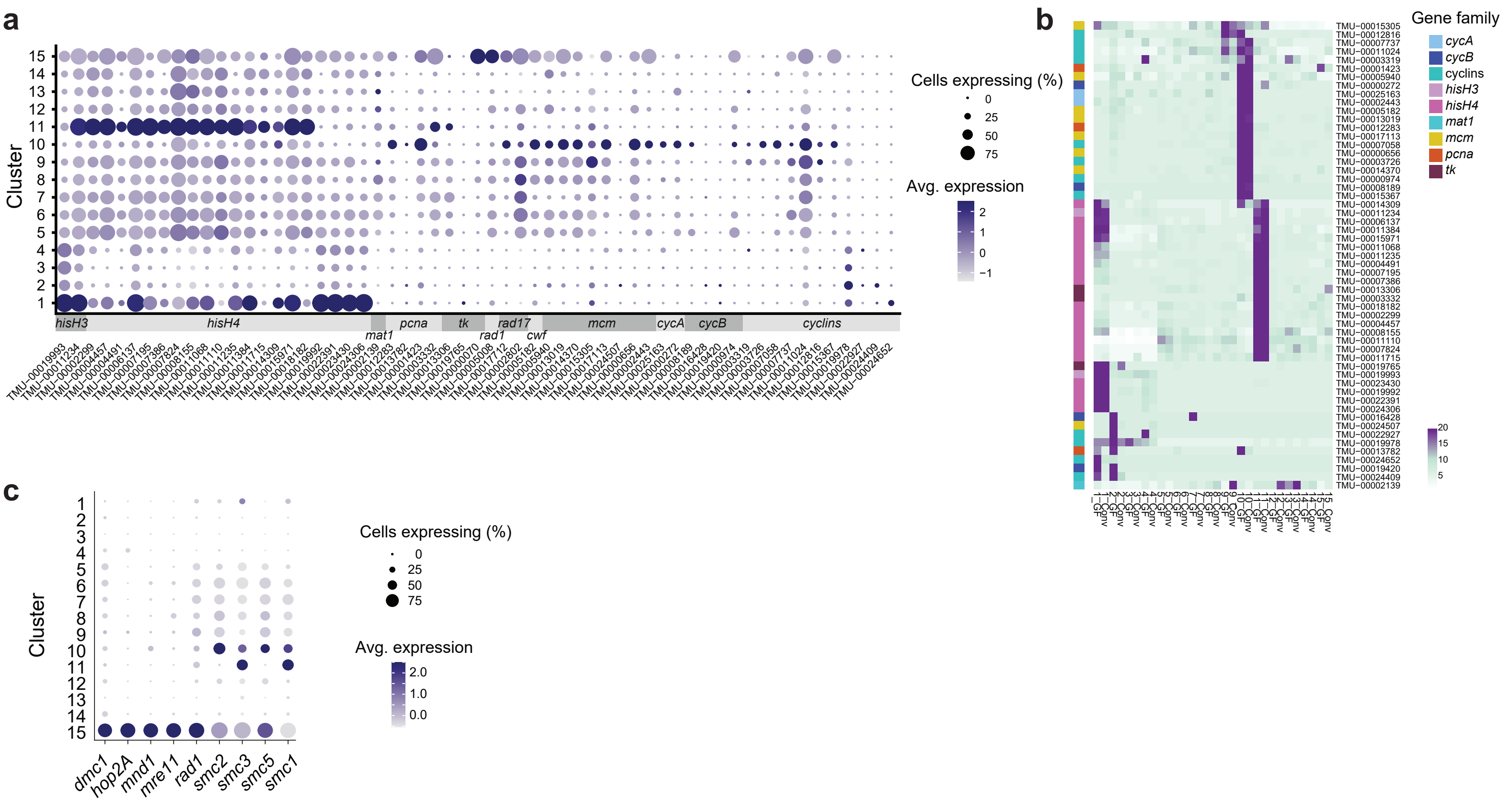

Supplementary Figure 6. Expression of cell cycle marker genes. **a**, Dotplot and **b**, heatmap depicting scaled read counts of genes known to be expressed during G1/S and G2 phases across each *Tmu* cluster. Colour blocks in **b** (*left*) indicate assigned gene function. Counts are separated by the host mouse. **c**, Expression of meiosis-specific genes.

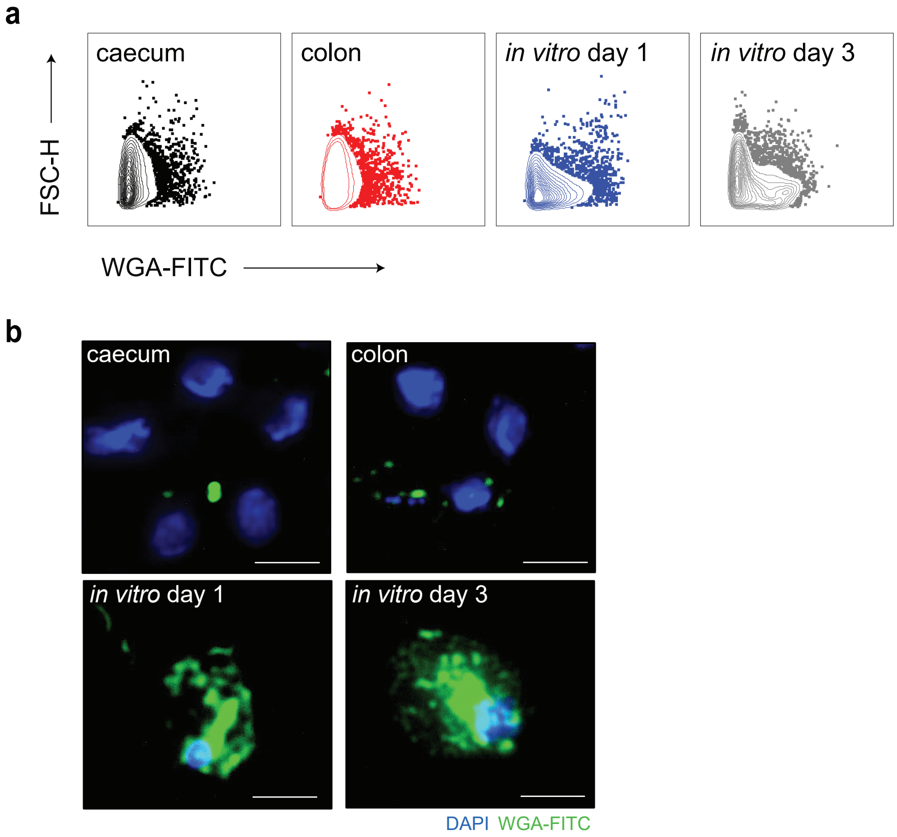

Supplementary Figure 7. WGA-staining of *Tmu* cells freshly isolated from mouse caeca or colons, or *in vitro* cultured for 1 to 3 days. **a,** Representative contour plots from FACS analysis of WGA-FITC stained protists. Events are gated on live single protists. n=3 animals or culture plates per group from four independent experiments. **b,** Cytospins of WGA-FITC (green) and DAPI (blue) stained protists. Representative images are shown at 63x magnification. Scale bars, 10 µm.

a

	KEGG-defined pathway	ECs	Control Day 2	Control Day 28	*Tmu*-infected Day 2	*Tmu*-infected Day 28
Amino acid metabolism	Valine, leucine and isoleucine degradation	7	ns	ns	ns	* (3)
Carbohydrate metabolism	Glycolysis / Gluconeogenesis	22	ns	ns	ns	*** (7)
Lipid metabolism	Fatty acid degradation	7	ns	ns	ns	** (4)
	Total significant ECs		433	166	349	349

b

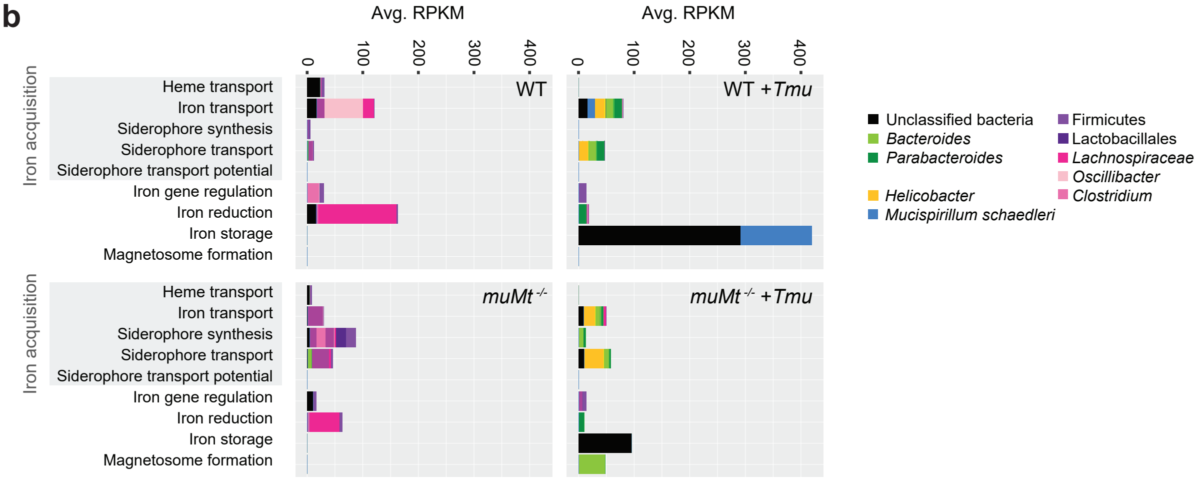

c

		Tmu infection				Host genotype (*muMt*-/- vs. WT)			
		Day 2		Day 28		Day 2		Day 28	
	Fe category	WT	*muMt*-/-	WT	*muMt*-/-	control	+*Tmu*	control	+*Tmu*
Iron acquisition	Iron transport (630)	*ns*	*ns*	*ns*	*ns*	*ns*	*ns*	*ns*	*ns*
Iron acquisition	Siderophore synthesis (799)	*ns*	*ns*	*ns*	*ns*	*ns*	*ns*	*ns*	*ns*
Iron acquisition	Siderophore transport (1067)	*ns*	*ns*	*** (162)	*** (97)	*ns*	*ns*	*** (36)	*ns*
Iron acquisition	Siderophore transport potential (134)	* (5)	** (12)	** (26)	*** (20)	*ns*	*ns*	*** (11)	*ns*
	Iron gene regulation (368)	*ns*	*ns*	** (56)	*ns*	*ns*	*ns*	*** (19)	*ns*
	Iron reduction (50)		*ns*	*ns*	*ns*	*ns*		*ns*	
	Iron storage (33)	*ns*	*ns*	** (10)	*ns*	*ns*	*ns*		
	Magnetosome formation (23)		*ns*	* (7)	** (6)	*ns*		*ns*	
		642	1980	7199	4110	6753	5382	1075	337

Supplementary Figure 8. Functional enrichment of bacterial gene expression due to protist colonization and host genotype. **a**, Metabolic pathway enrichment in caecal bacteria due to host genotype. **b**, RPKM of iron-related genes attributed to particular bacterial taxa at day 28 in WT or *muMt*-/- naïve and colonized mice. Colours reprsent taxa as indicated: black represents unclassified bacteria, greens are members of the Bacteroidota phylum, pinks and purples are Firmicutes, orange are Campilobacterota, blues are Deferribacterota. **c**, Enrichment of bacterial iron-related gene families due to protist colonization (*left*) and host genotype (*right*).
